# Enhanced skin cancer diagnosis using optimized CNN architecture and checkpoints for automated dermatological lesion classification

**DOI:** 10.1186/s12880-024-01356-8

**Published:** 2024-08-02

**Authors:** M Mohamed Musthafa, Mahesh T R, Vinoth Kumar V, Suresh Guluwadi

**Affiliations:** 1 Al-Ameen Engineering College (Autonomous), Erode, Tamil Nadu India; 2 Department of Computer Science and Engineering, JAIN (Deemed-to-be University), Bengaluru, 562112 India; 3 School of Computer Science Engineering and Information Systems, Vellore Institute of Technology University, Vellore, 632014 India; 4 Adama Science and Technology University, Adama, 302120 Ethiopia

**Keywords:** Skin lesion classification, Deep learning, Convolutional neural networks (CNN), Dermatoscopic images, Image Preprocessing, Data Augmentation

## Abstract

Skin cancer stands as one of the foremost challenges in oncology, with its early detection being crucial for successful treatment outcomes. Traditional diagnostic methods depend on dermatologist expertise, creating a need for more reliable, automated tools. This study explores deep learning, particularly Convolutional Neural Networks (CNNs), to enhance the accuracy and efficiency of skin cancer diagnosis. Leveraging the HAM10000 dataset, a comprehensive collection of dermatoscopic images encompassing a diverse range of skin lesions, this study introduces a sophisticated CNN model tailored for the nuanced task of skin lesion classification. The model’s architecture is intricately designed with multiple convolutional, pooling, and dense layers, aimed at capturing the complex visual features of skin lesions. To address the challenge of class imbalance within the dataset, an innovative data augmentation strategy is employed, ensuring a balanced representation of each lesion category during training. Furthermore, this study introduces a CNN model with optimized layer configuration and data augmentation, significantly boosting diagnostic precision in skin cancer detection. The model’s learning process is optimized using the Adam optimizer, with parameters fine-tuned over 50 epochs and a batch size of 128 to enhance the model’s ability to discern subtle patterns in the image data. A Model Checkpoint callback ensures the preservation of the best model iteration for future use. The proposed model demonstrates an accuracy of 97.78% with a notable precision of 97.9%, recall of 97.9%, and an F2 score of 97.8%, underscoring its potential as a robust tool in the early detection and classification of skin cancer, thereby supporting clinical decision-making and contributing to improved patient outcomes in dermatology.

## Introduction

Skin, the largest organ of the human body, serves as the primary barrier against environmental factors and plays a crucial role in protecting the body from various pathogens and harmful UV radiation. However, being the outermost layer, it is susceptible to a range of diseases, including various forms of skin cancer, which is among the most common types of cancer globally.

Skin diseases can range from benign conditions like acne or eczema to severe forms of cancer such as melanoma, basal cell carcinoma, and squamous cell carcinoma. Actinic keratoses (AK) and intraepithelial carcinoma (AKIEC) represent precancerous and early malignant growths, respectively, often arising from prolonged UV exposure. Basal cell carcinoma (BCC) is the most prevalent form of skin cancer, typically manifesting as pearly or waxy bumps, while melanoma (MEL) is an extremely aggressive malignancy arising from melanocytes, with potential for metastasis if not promptly treated. Benign keratosis-like lesions (BKL), dermatofibroma (DF), melanocytic nevi (NV), and vascular lesions present a spectrum of non-cancerous growths, each requiring careful assessment to rule out malignancy or address cosmetic concerns [1].

The differentiation between these conditions is vital, as it determines the treatment path and prognosis. The timely identification and treatment of AK and AKIEC can prevent progression to invasive carcinoma, while prompt intervention for BCC and MEL can mitigate the risk of metastasis and improve outcomes [2]. Furthermore, understanding the benign nature of BKL, DF, NV, and vascular lesions helps avoid unnecessary interventions while addressing patient concerns effectively.

Skin cancer specifically poses a significant health challenge due to its potential to spread to other body parts if not detected and treated early. Regular skin examinations, sun protection measures, and awareness of concerning changes in moles or skin lesions are essential for maintaining skin health and preventing the development or progression of skin cancer. Collaboration between patients, dermatologists, and healthcare providers is vital for comprehensive skin care, facilitating timely diagnosis, intervention, and long-term management strategies tailored to individual needs and risk factors [3].

Early detection of skin cancer significantly increases the chances of successful treatment and patient survival. Dermatologists traditionally diagnose skin conditions using visual examinations, which may be supplemented by dermoscopy, a non-invasive skin surface microscopy. However, these methods rely heavily on the clinician’s experience and can sometimes lead to subjective assessments.

The advent of machine learning and, more specifically, deep learning, has introduced new possibilities in the field of dermatology. By leveraging large datasets of dermatoscopic images, deep learning models, particularly Convolutional Neural Networks (CNNs), can be trained to recognize and classify various skin lesions with high accuracy [4]. This approach has the potential to support dermatologists in making more accurate diagnoses, leading to improved patient outcomes.

The motivation behind this research is to harness the power of deep learning to aid in the early detection and accurate classification of skin cancer, thereby addressing a critical gap in dermatological care. Despite advancements in medical technology, the rising incidence of skin cancer worldwide underscores the need for more accessible and reliable diagnostic tools. The ability of CNNs to analyze complex dermatoscopic images and learn from vast amounts of data presents an opportunity to develop an automated system that can assist even non-specialists in identifying potential skin cancers at an early stage [5]. This research is driven by the goal of democratizing access to high-quality diagnostic tools, especially in regions where dermatological expertise is scarce, and thereby contributing to the global fight against skin cancer.

This research paper presents a detailed study on the application of Convolutional Neural Networks to classify skin lesions using the HAM10000 dataset, which is a diverse collection of dermatoscopic images. The contributions of this research paper are:

- Introduce a sophisticated CNN model tailored for skin lesion classification, which significantly enhances diagnostic accuracy.
- Employ innovative data augmentation methods to address the issue of class imbalance within the HAM10000 dataset, enhancing the robustness of the model.
- Integrate Model Checkpoint callbacks to preserve the best model iteration during training, ensuring optimal performance in practical applications [6].

The next section provides a thorough review of the literature, highlighting previous studies on the use of deep learning in dermatology. The Methodology section details the dataset, the CNN architecture, the training process, and the evaluation metrics used in this study [7]. The results section presents the outcomes of the model training and validation, including performance metrics and a discussion on the model’s diagnostic accuracy. Finally, the paper concludes with a summary of the key findings and suggestions for further research in this rapidly evolving field.

## Related work

The fusion of artificial intelligence (AI) and deep learning has catalysed groundbreaking progress, significantly enhancing the precision of skin cancer diagnoses [8]. A notable study unveiled a convolutional neural network (CNN) that eclipsed dermatologists in identifying skin lesions, showcasing the model’s adeptness at distinguishing between benign and malignant lesions and illustrating AI’s potential to bolster dermatological clinical decision-making. Another study introduced an AI framework that amalgamates image analysis with demographic data, presenting a more holistic diagnostic approach that surpasses conventional image-centric models in predictive accuracy, highlighting the benefits of integrating diverse data types in AI-driven diagnostics.

### Traditional approaches

The traditional methods for diagnosing skin cancer primarily involve dermatological examinations and biopsies, where skilled dermatologists visually assess lesions and may take tissue samples for further histopathological analysis. These methods, while effective, rely heavily on the expertise and availability of specialists. This reliance can introduce variability in diagnosis accuracy and limits accessibility, particularly in underserved areas [9].

### Deep learning revolution

The integration of deep learning, particularly Convolutional Neural Networks (CNNs), into dermatological diagnostics marks a significant shift from traditional methods. CNNs excel in analyzing vast arrays of dermatoscopic images, automating the detection and classification processes [10]. These models can process detailed visual data at speeds and accuracies that challenge or even surpass human capabilities, transforming skin cancer diagnosis into a more efficient and standardized procedure.

### Challenges in deep learning

Deploying deep learning models in clinical settings is not without challenges. Key issues include the black-box nature of these models, which often lack transparency in their decision-making processes, making clinical acceptance difficult. Additionally, deep learning requires large, well-annotated datasets that are expensive and labor-intensive to compile. Models also face generalization challenges, where performance can drop when applied to new, unseen datasets, reflecting differences from the training data [11].

### Recent advances

To address these challenges, recent research has focused on enhancing the robustness and applicability of deep learning models. Approaches such as transfer learning have been employed to adapt pre-trained models to new tasks with less data, significantly improving performance. Innovations continue to emerge in model architecture and training methodologies, with some studies incorporating multiple data types—combining image data with patient demographic and clinical history—to refine diagnostic accuracy. These advancements are paving the way for more reliable and accessible automated diagnostic systems in dermatology [12]. Table 1 compares the methodologies and results to contextualize this study within the broader field.

**Table 1:** Recent studies

Study	Dataset Used	Accuracy	Remarks
Bhuvaneshwari Shetty et al. (2022) [13]	HAM10000 dataset (subset with augmentation)	95.18%	Focuses on detecting dangerous skin illnesses, particularly skin cancer.
Ahmad Hameed et al. (2023) [14]	MNIST HAM10000 dataset	95.2%	Addresses challenges in skin lesion detection and classification.
Md. Khairul Islam et al. (2021) [15]	HAM10000 dataset	96.10% in training, 90.93% in testing	Focus on skin cancer detection.
Ankita Pramanik & Rivu Chakraborty (2021) [16]	Dermatoscopic images from the Kaggle dataset archive	87.58%	Study on skin cancer detection in Caucasians in the USA.
Raut, Roshani et al. (2023) [17]	Dermoscopy images database	90.1%	AI integration for early melanoma identification.
Kalaycı, Serdar (2023) [18]	ISIC 2019 and ISIC 2020 datasets	AUC: 95.75%	Focus on early skin cancer detection.
Maaz Ul Amin et al. (2024) [19]	HAM10000 dataset, dataset of 3672 categorized pictures	84.27%	Study on global skin cancer concerns.
Aya Mostafa Mosa et al. (2022) [20]	Augmented dataset of 17,731 images + 3,800 from different types	Resnet18: 79.5%, Densnet121: 81.2%, InceptionV4: 82.6%	Focus on melanoma caused by UV exposure.
Mariame Oumoulylte et al. (2023) [21]	ISIC dataset	87%	Deep-learning models for binary classification of skin cancer.
Mohammad Atikur Rahman et al. (2024) [22]	Dataset of 2637 skin images	Optimized NASNet Mobile: 85.62%, NASNet Large: 83.98%	DCNN-based model for skin cancer classification.

## Methodology

**Fig. 1:**
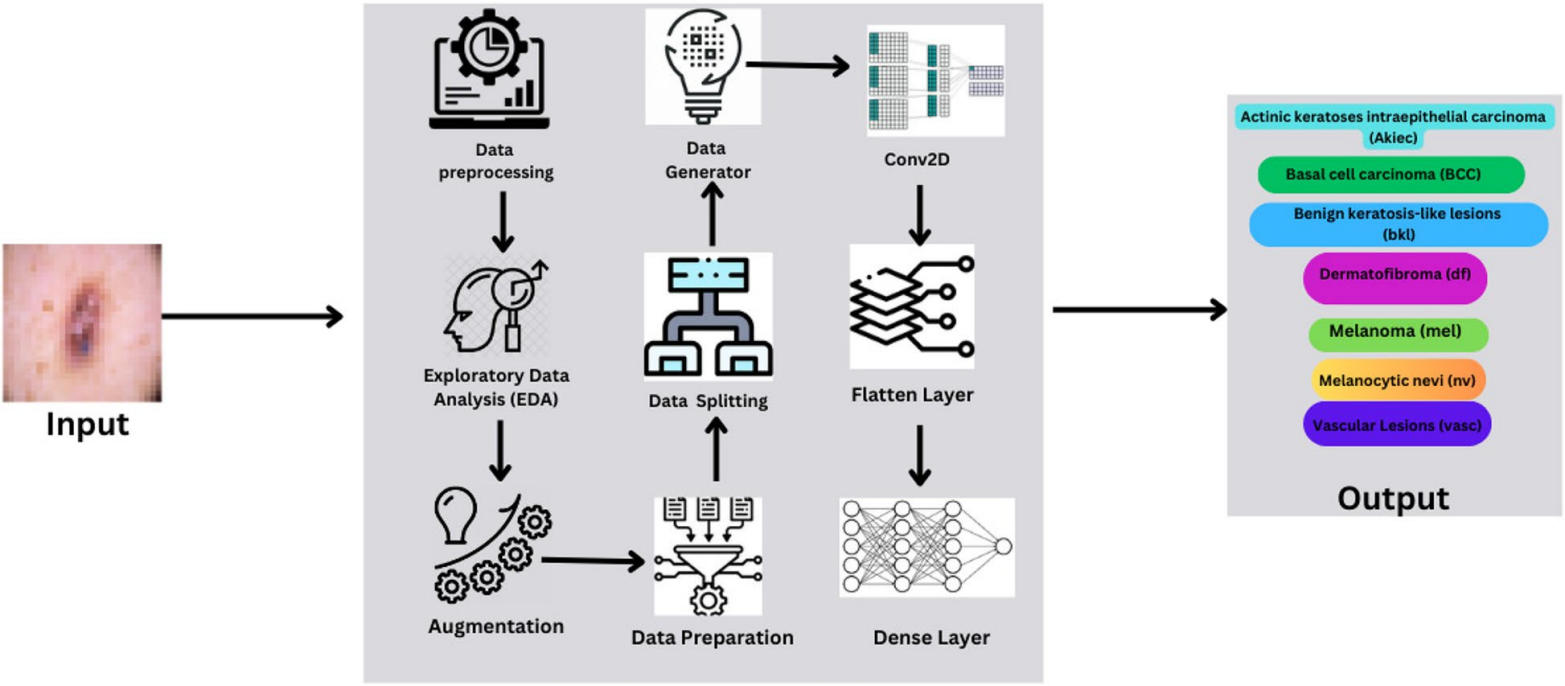
Workflow of the model

This section delineates the methodology adopted in this research to develop and evaluate the CNN model for classifying skin lesions using the HAM10000 dataset. It includes details on data preprocessing, model architecture, training procedures, and evaluation metrics. Figure 1 depicts the diagrammatic representation of the workflow of prosposed model.

### Dataset description

**Fig. 2:**
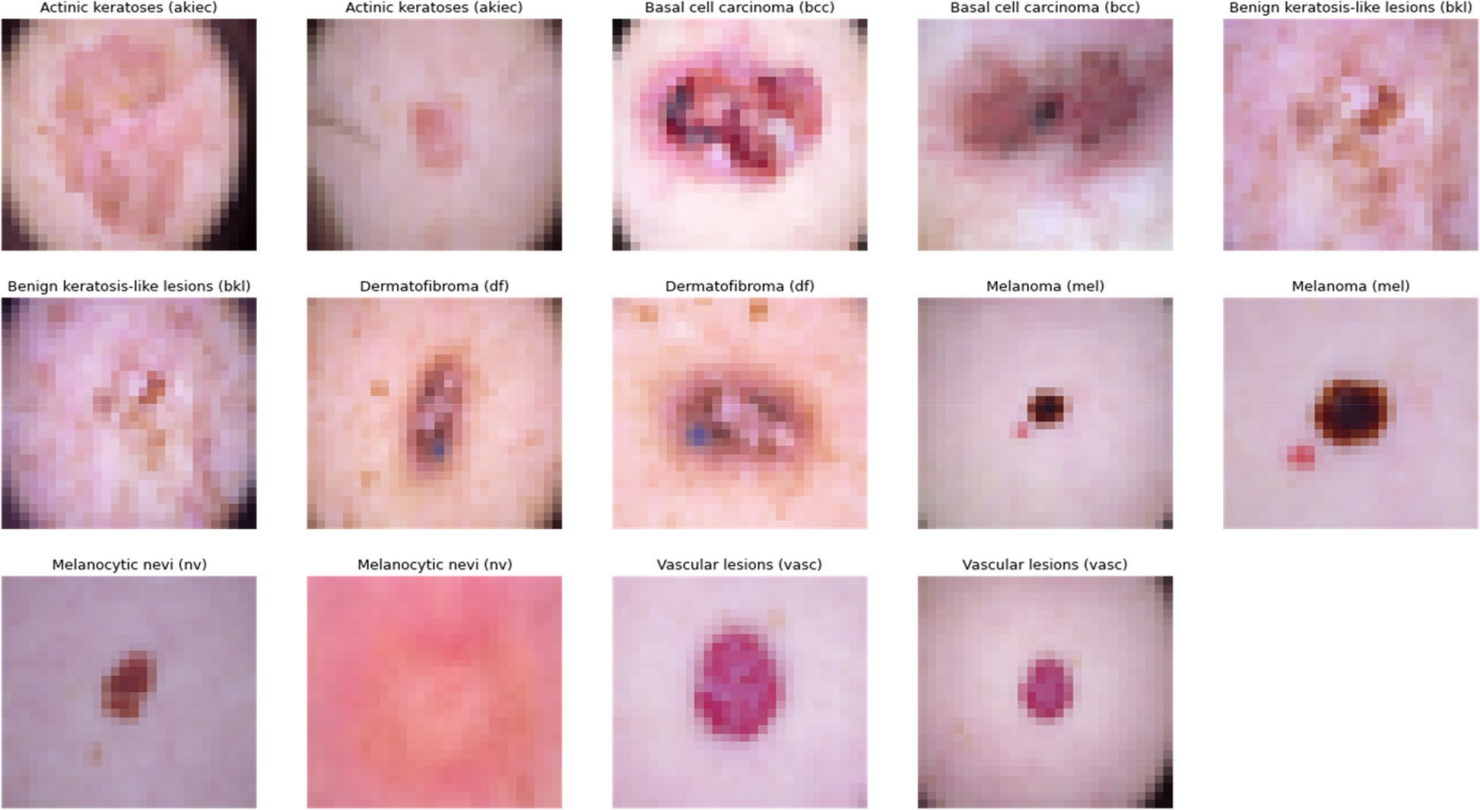
Input Images from dataset

The HAM10000 dataset, integral to dermatological advancements, encompasses over 10,000 dermatoscopic images, crucial for developing machine learning models for pigmented skin lesion diagnosis. It offers unparalleled diversity in skin tones, lesion types, and conditions, featuring seven distinct skin cancer categories: Actinic keratoses, intraepithelial carcinoma/Bowen’s disease, basal cell carcinoma, benign keratosis-like lesions, dermatofibroma, melanoma, and melanocytic nevi, alongside various vascular lesions. This diversity is vital for training robust algorithms. Over half of the lesions are histopathologically verified, with the remainder confirmed via follow-up, expert consensus, or confocal microscopy, ensuring data authenticity. The dataset’s utility is enhanced by metadata, such as lesion IDs, allowing for longitudinal lesion analysis, crucial for developing models that recognize temporal lesion changes. The HAM10000 dataset is a comprehensive, validated resource that propels the development of diagnostic tools in dermatology, offering a real-world dataset challenge to automate skin lesion analysis. Its depth, verification rigor, and inclusion of multiple cancer types make it an indispensable tool for improving dermatological diagnostic accuracy and reliability through machine learning. Figure 2 demonstrates different categories of skin cancer in the dataset.

### Image Preprocessing

Data preprocessing is a crucial step in preparing the dataset for training with a Convolutional Neural Network (CNN). It ensures that the input data is in a suitable format and is conducive to the learning process. In this study, the data preprocessing involved three main steps: image resizing, normalization, and data augmentation. It ensures that the model treats all features (pixels, in this case) equally.

#### Image resizing

To ensure that all images fed into the CNN have a consistent shape and size, each image in the HAM10000 dataset was resized to a standard dimension. This uniformity is essential because CNNs require a fixed size for all inputs to maintain a consistent architecture, especially when it comes to applying filters and pooling operations. Resizing images helps in creating a standardized input feature dimension, enabling the network to systematically extract features across all samples. The formula for resizing the image to a desired width ​ and height is presented in Eq. 1.

1 $${I}_{\mathrm {resized}}=\mathrm {resize}({I}_{\mathrm {original}},({W}_{\mathrm {new}},{H}_{\mathrm {new}})$$

Here, $\:{I}_{\text{original}}$ represent the original image with dimensions $\:{W}_{\text{original}}*{H}_{\text{original}}$

#### Normalization

Normalization refers to the process of scaling the pixel values of the images to a range of 0 to 1. This is achieved by dividing the pixel values by 255 (since pixel values range from 0 to 255) [23]. Normalizing the data is beneficial for the training process as it helps in speeding up the convergence by reducing the initial variability of the weights. When data is normalized, the gradients used in backpropagation are in a more manageable range, which helps in a smoother and faster optimization process, leading to more stable and quicker convergence. The formula for normalization is shown in Eq. 2.

2 $${I}_{\text{normalized}}\left(x,y\right)=\frac{{I}_{\text{original}}\left(x,y\right)}{255}$$

Where, $\:{I}_{\text{normalized}}$​ represent the normalized image and $\:{I}_{\text{original}}\left(x,y\right)$represent the pixel intensity value of the original image at location $\:(x,y).$

#### Data augmentation

**Fig. 3:**
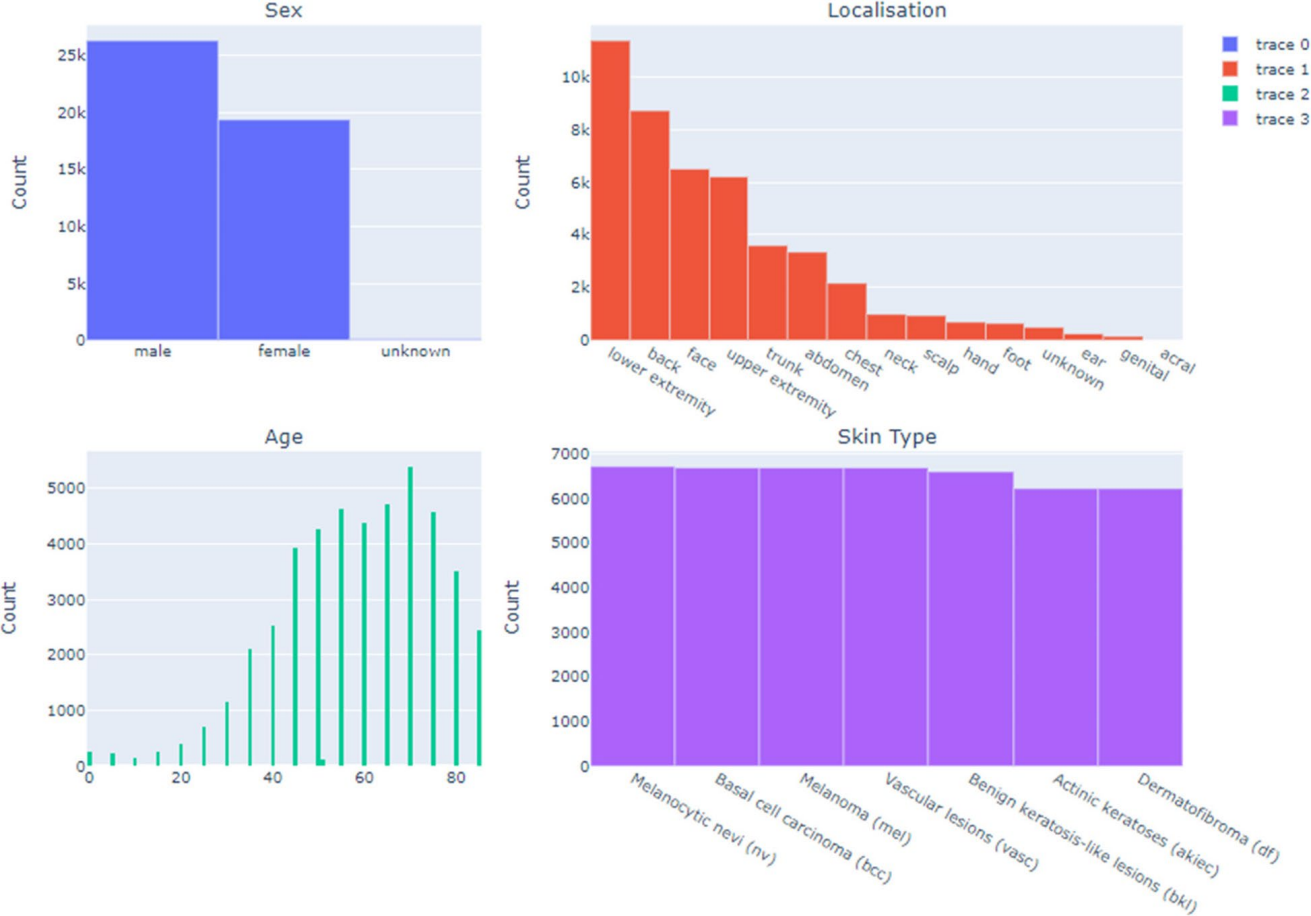
Dataset distribution after augmentation

Dataset augmentation is a critical strategy in machine learning that enriches training data diversity, particularly vital in image processing. By applying specific transformations like rotation, zooming, flipping, shearing, and brightness adjustment, this study simulate various real-world scenarios, thereby broadening the model’s exposure to potential data variations it might encounter post-deployment. These transformations not only enhance the model’s ability to generalize across new, unseen data, thereby improving its predictive accuracy, but also serve as a regularization technique, significantly reducing the risk of overfitting. Overfitting is a common challenge where the model learns noise alongside the underlying pattern, which can degrade its performance on novel data [1]. Through dataset augmentation, the model’s robustness and adaptability is ensured, reinforcing its performance stability across a spectrum of conditions, leading to more reliable and accurate predictions in practical applications. Together, these preprocessing steps form an integral part of the data preparation pipeline, setting a strong foundation for the subsequent training of the CNN model. Figure 3 showcases distribution of data after augmentation. The formula for data augmentation can vary depending on the specific transformation applied is represented in Eq. 3.

3 $${I}_{\text{augmented}}=\text{transform}\left({I}_{\text{original}},T\right)$$

Where, $\:{I}_{\text{augmented}}\:$represent the augmented image obtained from the original image $\:{I}_{\text{original}}$ by applying transformations *T*.

### Model architecture

**Fig. 4:**
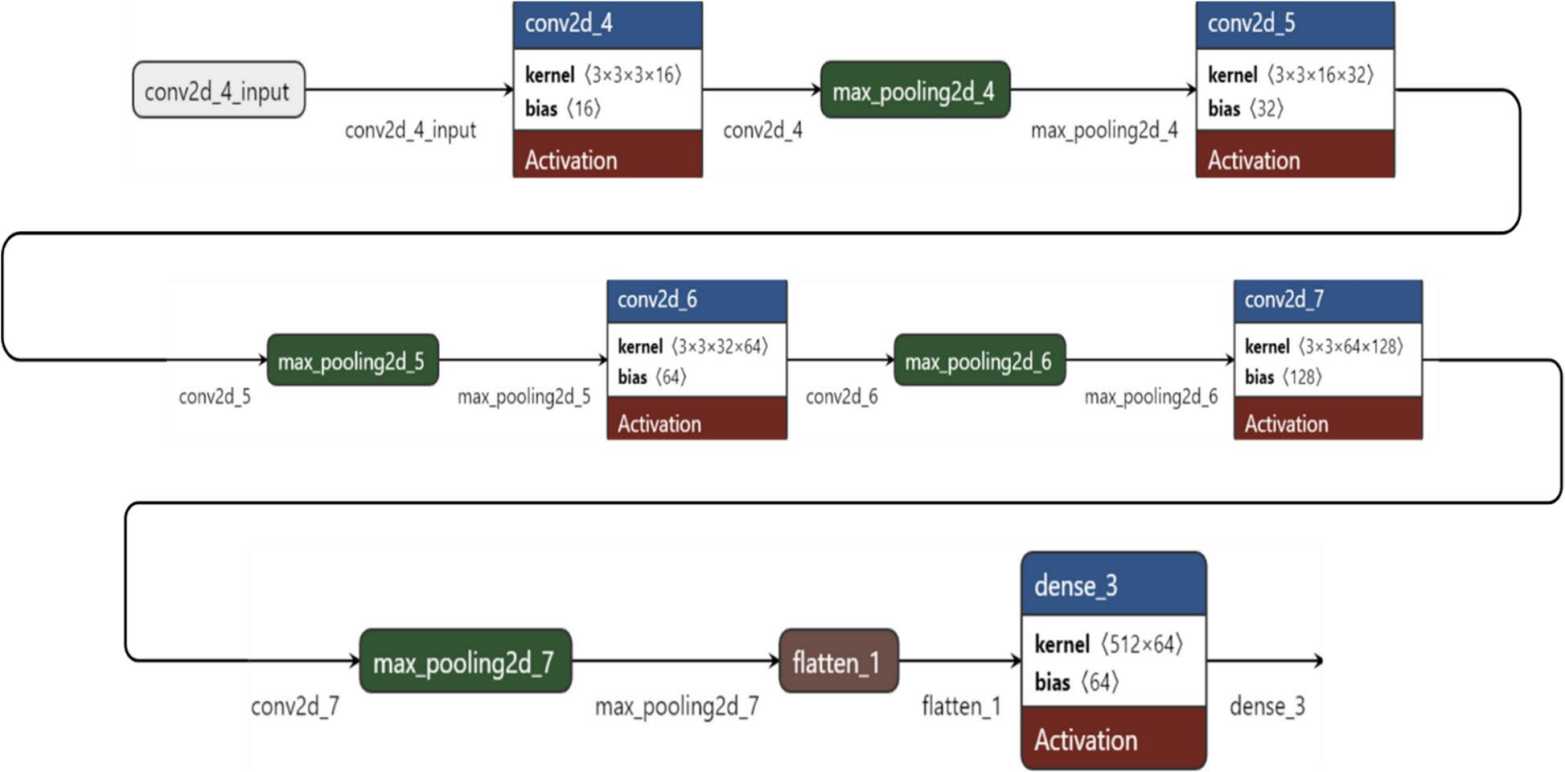
Layer wise network architecture diagram

A Convolutional Neural Network (CNN) is a deep neural network architecture used for analysing visual imagery, known for its efficacy in various computer vision tasks. At its core, a CNN employs convolutional layers where filters or kernels extract specific features from images, with the ReLU activation function introducing necessary non-linearities. Following these, pooling layers, typically max pooling, reduce the feature maps’ spatial dimensions, aiding in computational efficiency and spatial invariance. The network then utilizes dense or fully connected layers, where the data, now flattened, undergoes high-level reasoning through connections that encompass weights and further activation functions. The culmination is an output layer where a SoftMax activation function translates the final layer’s outputs into probability distributions across multiple classes. In training, the Adam optimizer is favoured for its adaptive learning rate, working alongside a sparse categorical cross entropy loss function to iteratively adjust the network’s weights, thereby minimizing the discrepancy between the model’s predictions and the actual data labels. This intricate orchestration of layers and functions allows CNNs to adeptly learn and identify patterns in visual data, making them particularly suitable for tasks like skin lesion classification where distinguishing nuanced visual features is paramount [24]. Table 2 provides the layer type, output shape, and the number of parameters for each layer in the model while Fig. 4 illustrates the network architecture of the model.

**Table 2:** Parameters for training CNN Model

Layer (type)	Output Shape	Param #
conv2d_4	(None, 28, 28, 16)	448
max_pooling2d_4	(None, 14, 14, 16)	0
conv2d_5	(None, 14, 14, 32)	4640
max_pooling2d_5	(None, 7, 7, 32)	0
conv2d_6	(None, 7, 7, 64)	18,496
max_pooling2d_6	(None, 4, 4, 64)	0
conv2d_7	(None, 4, 4, 128)	73,856
max_pooling2d_7	(None, 2, 2, 128)	0
flatten_1	(None, 512)	0
dense_3	(None, 64)	32,832
dense_4	(None, 32)	2080
dense_5	(None, 7)	231

The architecture of this model, comprising sequential convolutional and pooling layers followed by a dense output layer, is strategically designed for the effective classification of skin lesions. Starting with convolutional layers (conv2d_4, conv2d_5, and conv2d_7) that progressively increase in filter depth, the model captures increasingly complex features from the dermatoscopic images, essential for distinguishing subtle differences between various skin lesion types. Each convolutional layer is paired with a max pooling layer (max_pooling2d_4, max_pooling2d_5, max_pooling2d_7), which reduces the spatial dimensions of the feature maps, thus lowering computational demands and focusing the model on dominant features critical for accurate classification. The ‘flatten_1’ layer transitions these 2D feature maps into a 1D vector, preparing them for the final classification performed by the dense layer (dense_3). This architecture not only aligns with proven image classification principles but also balances computational efficiency with high diagnostic performance, making it suitable for real-world clinical applications where resources may be limited and high accuracy is paramount.

The Convolutional Neural Network (CNN) architecture is designed with a strategic layering of convolutional, pooling, fully connected, and dropout layers, each serving a distinct function in processing and classifying visual data, particularly for tasks like skin lesion classification.

#### Convolutional layers

These layers are the building blocks of the CNN, responsible for detecting patterns such as edges, textures, and more complex shapes within the images. The depth and number of filters in these layers are chosen to capture a broad spectrum of features without overwhelming the model’s capacity. This Eq. 4 represents the mathematical operation of convolution, where *f* is the input image and *g* are the filter (kernel) applied to extract features.

4 $$\left(f*g\right)\left(t\right)={\int\:}_{-{\infty\:}}^{{\infty\:}}f\left({\uptau\:}\right)g\left(t-{\uptau\:}\right)d{\uptau\:}$$

The depth (number of filters) and size of the filters in these layers are meticulously chosen to optimize the model’s ability to detect a wide range of features without overburdening its computational capacity.

Activation Function: Following the convolution operation, an activation function, typically the Rectified Linear Unit (ReLU), is applied to introduce non-linearity into the network. This step is essential as it allows the model to learn and represent more complex patterns in the data. Equation 5 defines ReLU, which introduces non-linearity into the network by outputting the input if it is positive and zero otherwise.

5 $$f\left(x\right)=\text{max}\left(0,x\right)$$

#### Pooling layers

After each convolutional layer, pooling layers reduce the dimensionality of the data, summarizing the features extracted while retaining the most salient information. This reduction is crucial for minimizing computational load and enhancing the model’s focus on essential features. Equation 6 describes the max-pooling operation, which down samples the feature maps generated by convolutional layers.

6 $$max\ pooling (x,y)=max_{i\in\ R}x[i,y]$$

#### Flatten layer

This layer transitions from the 2D output of the pooling layers to a 1D vector. It’s a crucial step, transforming the processed image data into a format suitable for the fully connected layers, enabling the network to interpret the extracted features comprehensively. Equation 7 reshapes the output of the convolutional layers into a one-dimensional vector, which serves as input to the fully connected layers.

7 $$f\left({x}_{ij}\right)={x}_{k}$$

#### Fully connected

Following the extraction and down-sampling of features, the network transitions to fully connected layers. In these layers, neurons have connections to all activations in the previous layer, as opposed to convolutional layers where neurons are connected to only a local region of the input. This architecture allows the network to combine features learned across the entire image, enabling high-level reasoning and classification, and is computed by using the formula depicted in Eq. 8.

8 $$y=f\left(Wx+b\right)$$

#### Dropout layers

To mitigate the risk of overfitting, dropout layers are incorporated, randomly disabling a fraction of the neurons during training. This forces the network to learn more robust features that are not reliant on a small set of neurons, enhancing its generalization capability. To adjust for dropout at training time, activations at test time are scaled by the dropout probability p as in Eq. 9.

9 $$\stackrel{\sim}{x}=\frac{x}{p}\cdot\:\text{Bernoulli}\left(p\right)$$

#### Batch normalization

Batch normalization standardizes the inputs of each layer, improving the stability and speed of the network’s learning phase. The formula in Eq. 10 scales the activations of a layer by the learned parameter $\:{d}_{i}$​. Equation 11 calculates the batch-normalized output for each activation and Eq. 12 adjusts the activations during training and testing phases to maintain consistency.

10 $${a}_{i}={a}_{i}\cdot\:{d}_{i}$$

11 $$\hat{{x}_{i}}=\frac{{x}_{i}-{\mu\:}}{\sqrt{{{\sigma\:}}^{2}+{\in}}}$$

12 $${a}_{i}^{test}=p\cdot\:{a}_{i}^{train}$$

#### Output layer

The final layer in a CNN is the output layer, where a SoftMax activation function is typically used for multi-class classification tasks. The SoftMax function converts the output scores from the final dense layer into probability values for each class. In the context of skin lesion classification, it provides the probabilities of an image belonging to each of the seven lesion categories. The SoftMax function, expressed in Eq. 13 converts the logits (raw predictions) from the last dense layer into a probability distribution across the classes. This equation represents the partial derivative of the SoftMax output $\:{S}_{j}$ with respect to the input *xj*​, where $\:{\delta\:}_{ij}$is the Kronecker delta.

13 $$\frac{\partial\:{S}_{i}}{\partial\:{x}_{j}}={S}_{i}\left({\delta\:}_{ij}-{S}_{j}\right)$$

The CNN architecture adeptly learns to identify and classify complex patterns in visual data, making it an ideal choice for medical image analysis, including skin lesion classification. Algorithm 1 encapsulates a comprehensive approach to classify dermatological conditions using CNNs, addressing key aspects like data preprocessing, augmentation, model construction, training, and evaluation.

**Figure:**
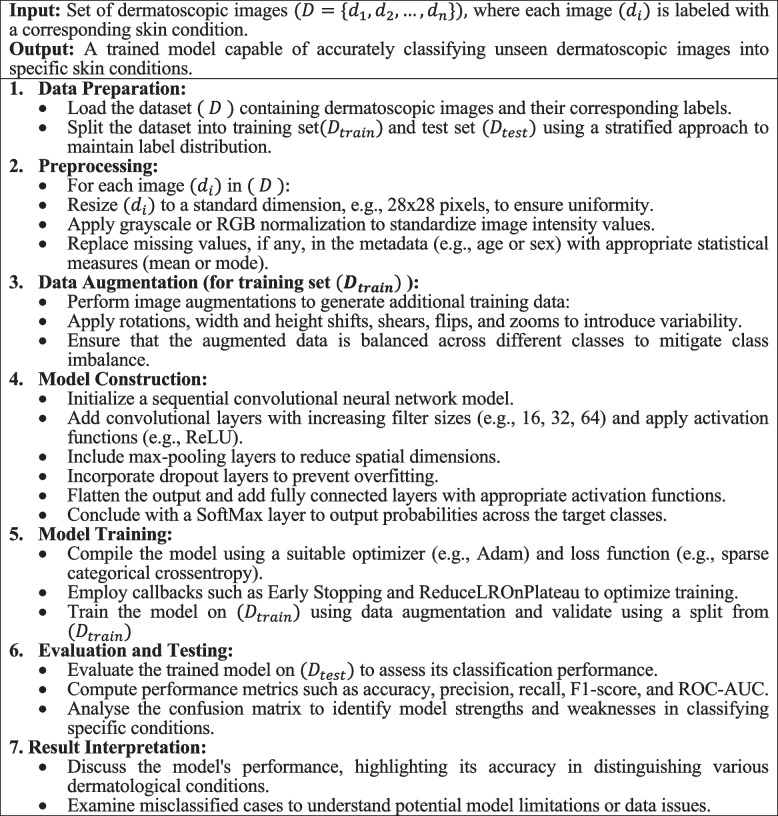
** Algorithm 1.**  Enhanced image classification for dermatological conditions

### Training procedure

Training the model is a delicate balance of maximizing learning while avoiding overfitting, with various strategies employed to achieve this equilibrium.

#### Data splitting

The division of data into training, validation, and test sets is pivotal. The training set is the model’s learning ground, while the validation set guides the tuning of hyperparameters and early stopping criteria. The test set remains untouched until the final evaluation, ensuring an unbiased assessment of the model’s generalization capabilities [25].

#### Optimization and loss

**Fig. 5:**
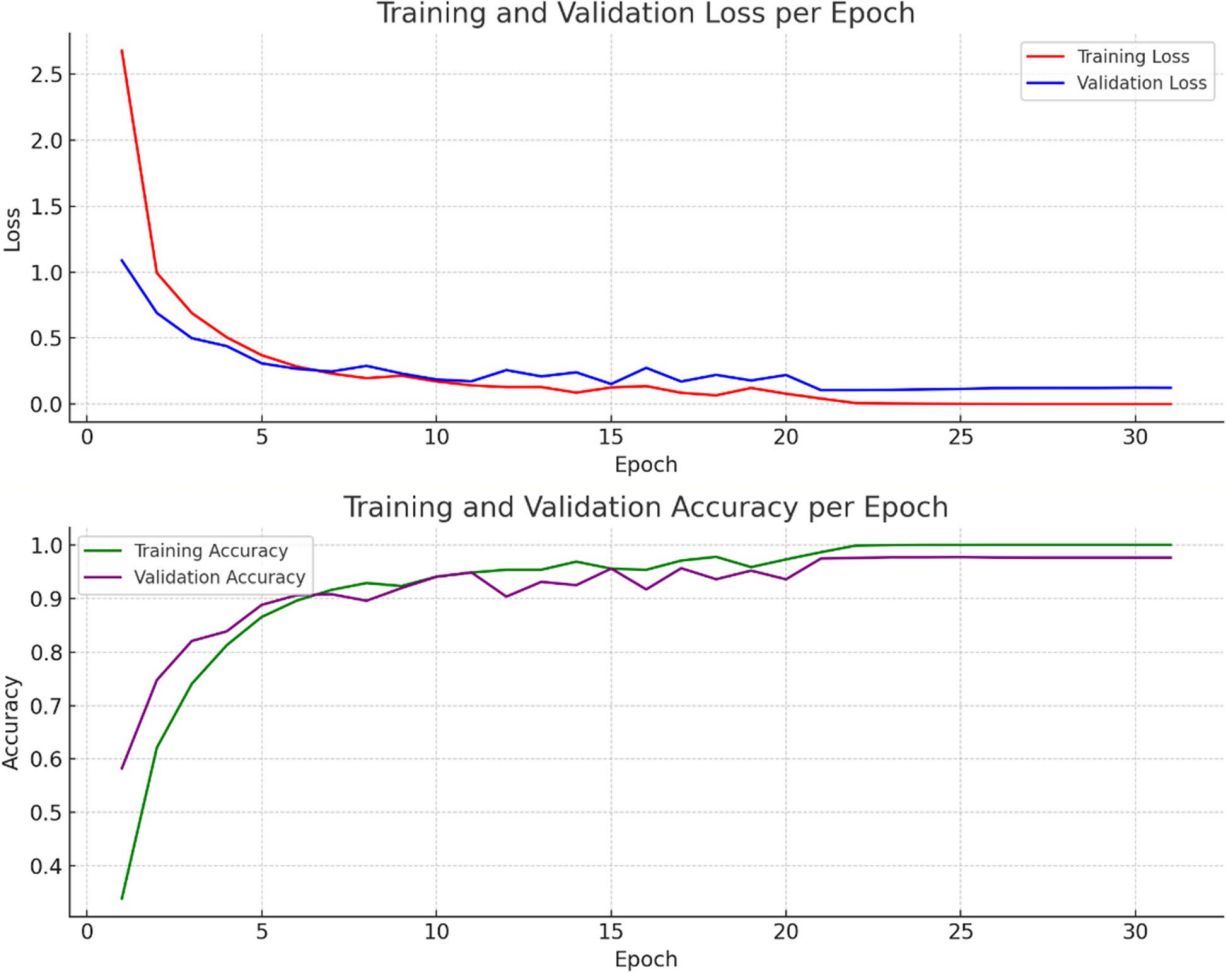
Training and validation loss and accuracy per epochs

The Adam optimizer is a choice grounded in its adaptive learning rate mechanism, which tailors the update magnitude for each weight, optimizing the learning process. The categorical cross-entropy loss function quantifies the disparity between the predicted probabilities and the actual distribution, guiding the model toward more accurate predictions. Figure 5 Visualizes the model’s increasing accuracy through training phases, emphasizing its learning efficiency. Equation 14 adjusts the learning rate using the ReduceLRonPlateau method based on the old learning rate and a reduction factor, Eq. 15 updates the weight in the Adam optimizer by incorporating the first and second moment vectors, a small constant epsilon, and the learning rate and Eq. 16 computes the loss in classification tasks by summing the negative logarithm of predicted probabilities weighted by true labels.

14 $${{\upeta\:}}_{\text{new}}=\text{ReduceLRonPlateau}\left({{\upeta\:}}_{\text{old}},\text{factor}\right)$$

15 $${W}_{t+1}={W}_{t}-{v}_{t}+{\in}{\upeta\:}{m}_{t}$$

16 $$L=-{\sum\:}_{i}{y}_{i}\text{log}\left({p}_{i}\right)$$

While initial model training was originally planned for 50 epochs to thoroughly optimize learning and parameter adjustments, Fig. 5 reveals signs of overfitting emerging after 20 epochs. Despite this, in the study it was chosed to continue training for the full 50 epochs to fully exploit the model’s learning potential under controlled conditions, facilitating fine-tuning of parameters like learning rate and regularization techniques. To counteract the observed overfitting risk post-20 epochs, several strategies were implemented: Early Stopping was employed to halt training when validation loss ceased to improve, preventing the model from memorizing noise; Model Checkpointing saved the best-performing model based on validation set performance; and regularization techniques such as dropout and L2 regularization were utilized to mitigate overfitting by penalizing complex models. These efforts ensured this model’s resilience and generalizability despite the extended training period. Detailed performance metrics in the results section underscore the effectiveness of these strategies in combating overfitting and optimizing overall model performance.

#### Callbacks

Early Stopping monitors the validation loss, ceasing training when improvements halt, thereby averting overfitting. Model Checkpoint saves the model at its peak performance on the validation set, ensuring that the best version is retained for evaluation and future application.

### Evaluation metrics

To evaluate the effectiveness and robustness of the deep learning model, various metrics were employed:

#### Exploratory Data Analysis (EDA)

A comprehensive exploratory data analysis (EDA) was conducted on the HAM10000 dataset to extract critical insights for the development of a Convolutional Neural Network (CNN) aimed at diagnosing skin cancer. The analysis encompassed an examination of the distribution of patient demographics and lesion localizations, facilitating a broad representation of cases to augment the model’s generalizability. Age distribution and lesion type frequencies were scrutinized to strategically address the dataset’s class imbalance through targeted data augmentation techniques. An in-depth visual assessment of dermatoscopic images from each lesion category informed the CNN architecture design and data preprocessing methods. This extensive EDA process yielded essential statistical insights, guiding a data-driven model development approach that ensures accuracy, equity, and generalizability across varied patient demographics and lesion types, laying a robust foundation for the modeling endeavors that followed.

#### Accuracy

This metric measures the overall correctness of the model, calculated as the ratio of correctly predicted observations to the total observations [26]. It provides a high-level overview of the model’s performance and is calculated by using the formula presented in Eq. 17.

17 $$Accuracy=\frac{TP+TN}{TP+TN+FP+FN\:}$$

#### Precision

Precision, or the positive predictive value, indicates the ratio of correctly predicted positive observations to the total predicted positives. It’s crucial for scenarios where the cost of false positives is high [27]. Equation 18 illustrates the formula to calculate precision.

18 $$Precision=\frac{TP}{TP+FP}$$

#### Recall (sensitivity)

Recall measures the ratio of correctly predicted positive observations to all actual positives [28]. It’s particularly important in medical diagnostics, where failing to detect a condition could have serious implications. Recall is calculated using the formula depicted in Eq. 19.

19 $$Recall=\frac{TP}{TP+FN}$$

#### F1-score

The F1-score is the harmonic mean of precision and recall, providing a single metric that balances both [29]. In addition to accuracy, precision, and recall, the F1 score serves as a critical indicator of model performance, especially in the domain of medical diagnostics. The F1 score, which is the harmonic mean of precision and recall, provides a single metric that balances the trade-off between these two critical aspects. This is particularly important in medical image classification where both false positives and false negatives carry significant consequences. A high F1 score indicates not only that the model accurately identifies a high number of relevant instances but also that it minimizes the number of incorrect classifications on crucial negative cases. This balance is essential for ensuring reliable clinical decisions based on the model’s predictions. It’s useful when it is needed to balance precision and recall and is depicted by a formula in Eq. 20. This metric is particularly advantageous in scenarios where an uneven class distribution might render other metrics less informative. For instance, in cases where the prevalence of positive class (skin lesions that are cancerous) is much lower, precision and recall individually may not adequately reflect the performance nuances of the diagnostic model. By combining these metrics, the F1 score provides a more robust indicator of the model’s effectiveness across various classes, thereby supporting its utility in clinical applications where accuracy and reliability are paramount.

20 $$F1=2\times\:\frac{Precision\times\:Recall}{Precision+Recall}$$

#### Confusion matrix

This matrix provides a detailed breakdown of the model’s predictions, showing the correct and incorrect predictions across different classes.

#### ROC curve and AUC

The Receiver Operating Characteristic (ROC) curve and the Area Under the Curve (AUC) provide insights into the model’s ability to distinguish between classes. A higher AUC indicates a better performing model. Equation 21 calculates the True Positive Rate (TPR), also known as Sensitivity or Recall. Similarly, Eq. 22 calculates the False Positive Rate (FPR) and Eq. 23 is used to calculate the Area Under the Curve (AUC) for a Receiver Operating Characteristic (ROC) curve.

21 $$\text{TPR}=\frac{\text{True Positives}}{\text{True Positives + False Negatives}}$$

22 $$\text{FPR}=\frac{\text{False Positives}}{\text{False Positives + True Negatives}}$$

23 $$\text{AUC}={\int\:}_{0}^{1}\text{TPR}\hspace{0.17em}\text{d(FPR)}$$

Through this detailed methodology, the research aims to forge a CNN model that is not just statistically accurate but also clinically viable, providing a tool that can potentially revolutionize the early detection and classification of skin lesions.

### Model training and validation

The training process involved feeding the pre-processed images and their corresponding labels into the CNN, allowing the network to iteratively learn from the data through backpropagation and gradient descent. The model’s parameters were updated to minimize the loss function, which quantifies the difference between the predicted and actual labels. To prevent overfitting, techniques such as dropout and early stopping were employed, wherein the training is halted when the validation loss ceases to decrease, ensuring the model’s generalizability to unseen data.

**Fig. 6:**
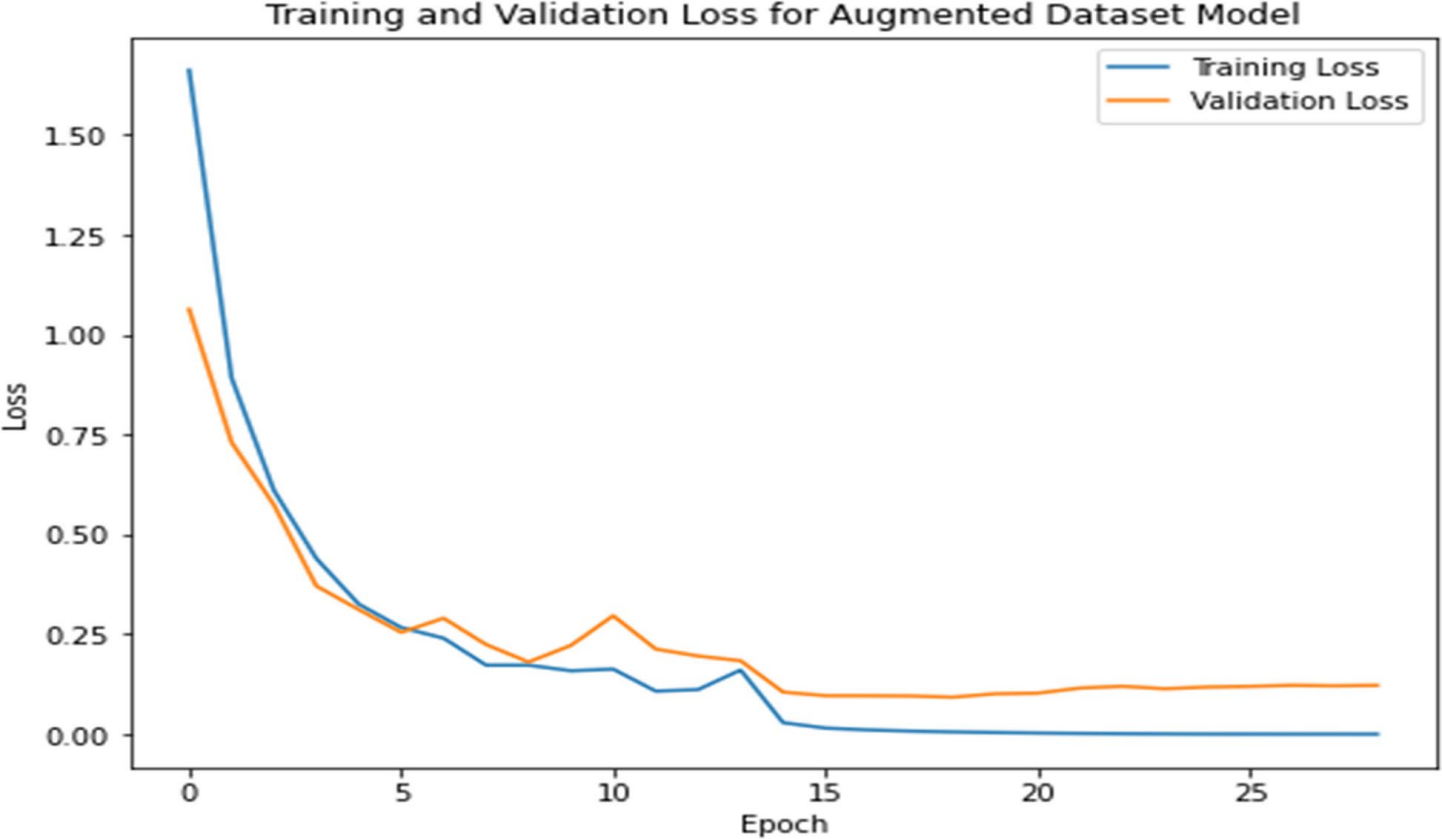
Training and validation loss graph for augmented dataset

During the model’s training, validation played a pivotal role, offering a lens through which the model’s performance on a subset of data, unseen during the training, was monitored. This phase was instrumental in identifying overfitting instances and guiding the hyperparameter tuning process. Hyperparameter tuning, an essential facet of the training regimen, involved the careful selection and adjustment of various parameters, including the learning rate, batch size, number of epochs, and architecture-specific settings such as the number and size of filters in convolutional layers, and the dropout rate. The learning rate, a critical hyperparameter, dictates the step size at each iteration while moving toward a minimum of the loss function, requiring a delicate balance to avoid underfitting or missing the minimum. Batch size influences the model’s convergence speed and generalization capabilities, with smaller batches offering a regularizing effect and larger batches providing computational efficiency. Through a combination of techniques like grid search, random search, or more sophisticated methods like Bayesian optimization, these hyperparameters were iteratively adjusted, with the model’s performance on the validation set serving as the benchmark for selecting the optimal combination, culminating in the selection of the best model iteration for evaluation on the test set [30]. Figure 6 tracks the model’s learning progress over epochs, highlighting improvements and stabilization in loss values.

## Experimentation and results

**Fig. 7:**
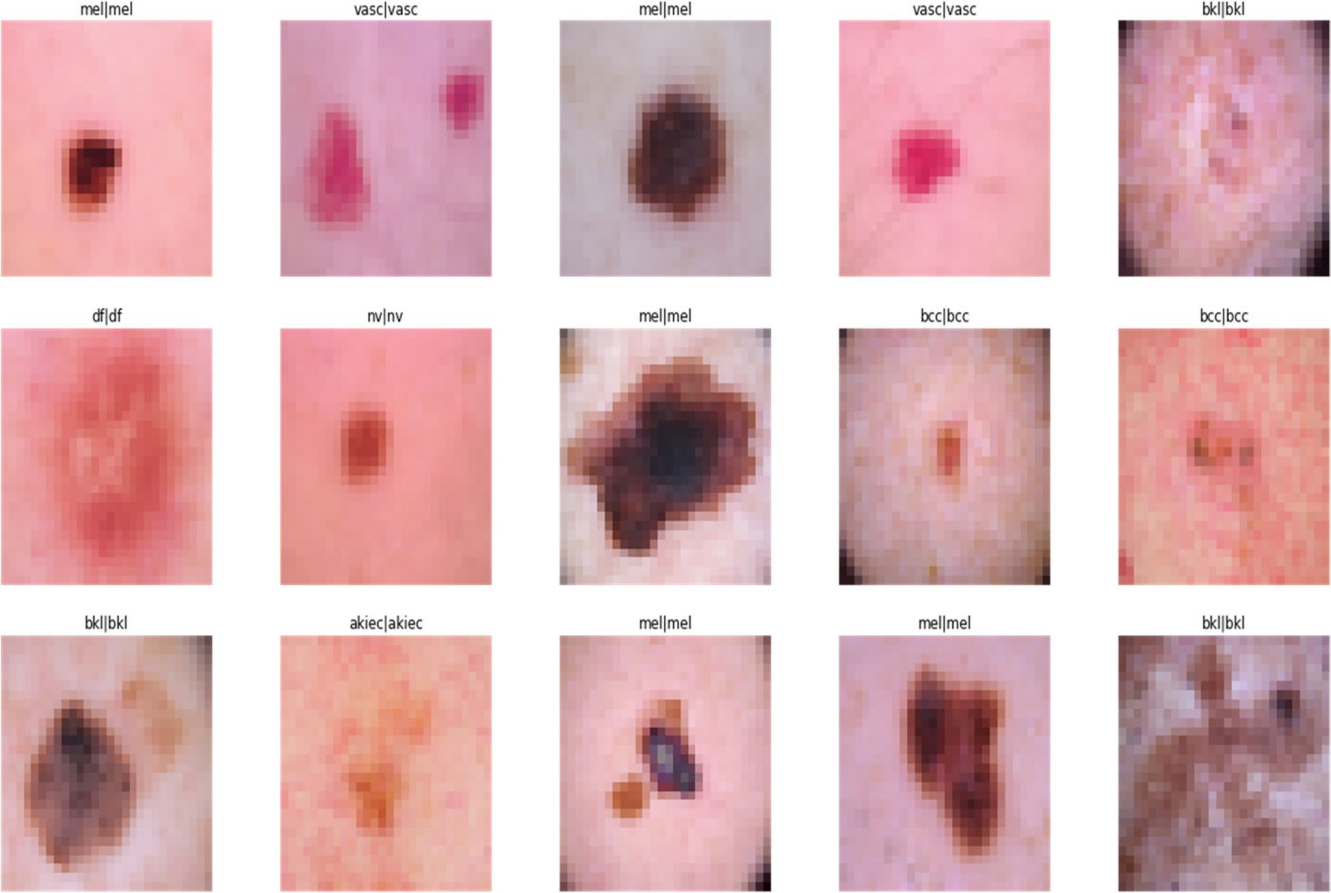
Predicted label with images

In this study an experimental framework to evaluate the performance of the Convolutional Neural Network (CNN) on the HAM10000 dataset is designed, a comprehensive collection of dermatoscopic images of skin lesions. The dataset was split into training (80%), validation (10%), and test (10%) sets to ensure a robust evaluation. The CNN architecture was constructed with multiple convolutional and pooling layers, followed by fully connected layers, and a SoftMax output layer to classify the images into seven distinct skin lesion categories. Figure 7 showcases the detection of skin cancer for different categories of skin cancer.

To enhance the model’s ability to generalize and to mitigate the risk of overfitting due to the limited size of the dataset, author’s applied extensive data augmentation techniques including rotations, zooming, and flipping. This approach not only expanded training dataset but also introduced a diversity of image perspectives, simulating real-world variations. Alongside augmentation, each image was resized and normalized to maintain consistency and to facilitate faster convergence during the training process.

The CNN was trained using the Adam optimizer and a sparse categorical crossentropy loss function. In this study early stopping and model checkpoint callbacks is employed to prevent overfitting and to save the best model during training. The training was conducted over numerous epochs, with real-time monitoring of performance metrics on both training and validation sets to ensure the model’s learning efficacy and generalization capability.

Upon completion of the training, the model demonstrated remarkable performance on the test set, achieving an overall accuracy of 97.858%. This high accuracy underscores the CNN’s ability to effectively differentiate between various types of skin lesions. The precision, recall, and F1-scores across different categories further validated the model’s robustness, with most classes showing scores above 90%, reflecting the model’s precision and reliability in classification.

**Fig. 8:**
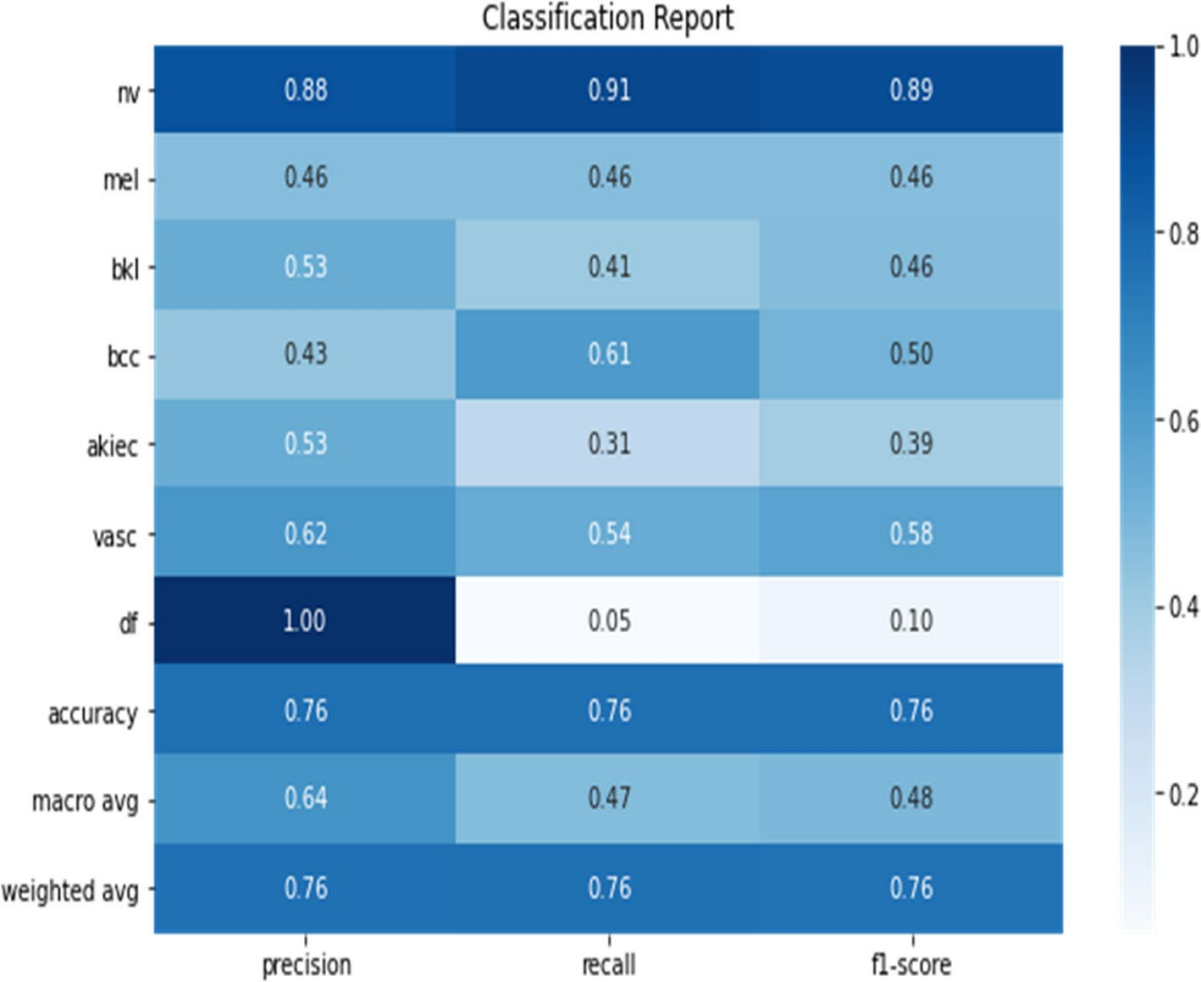
Classification report

The classification report provided detailed insights into the model’s performance across individual classes. Notably, the model excelled in identifying Melanoma (mel) and Basal cell carcinoma (bcc), which are critical for early cancer detection. The high F1-scores in these categories indicate a balanced precision-recall trade-off, crucial in medical diagnostics where both false negatives and false positives have significant implications. Figure 8 illustrates the classification report of the model.

**Fig. 9:**
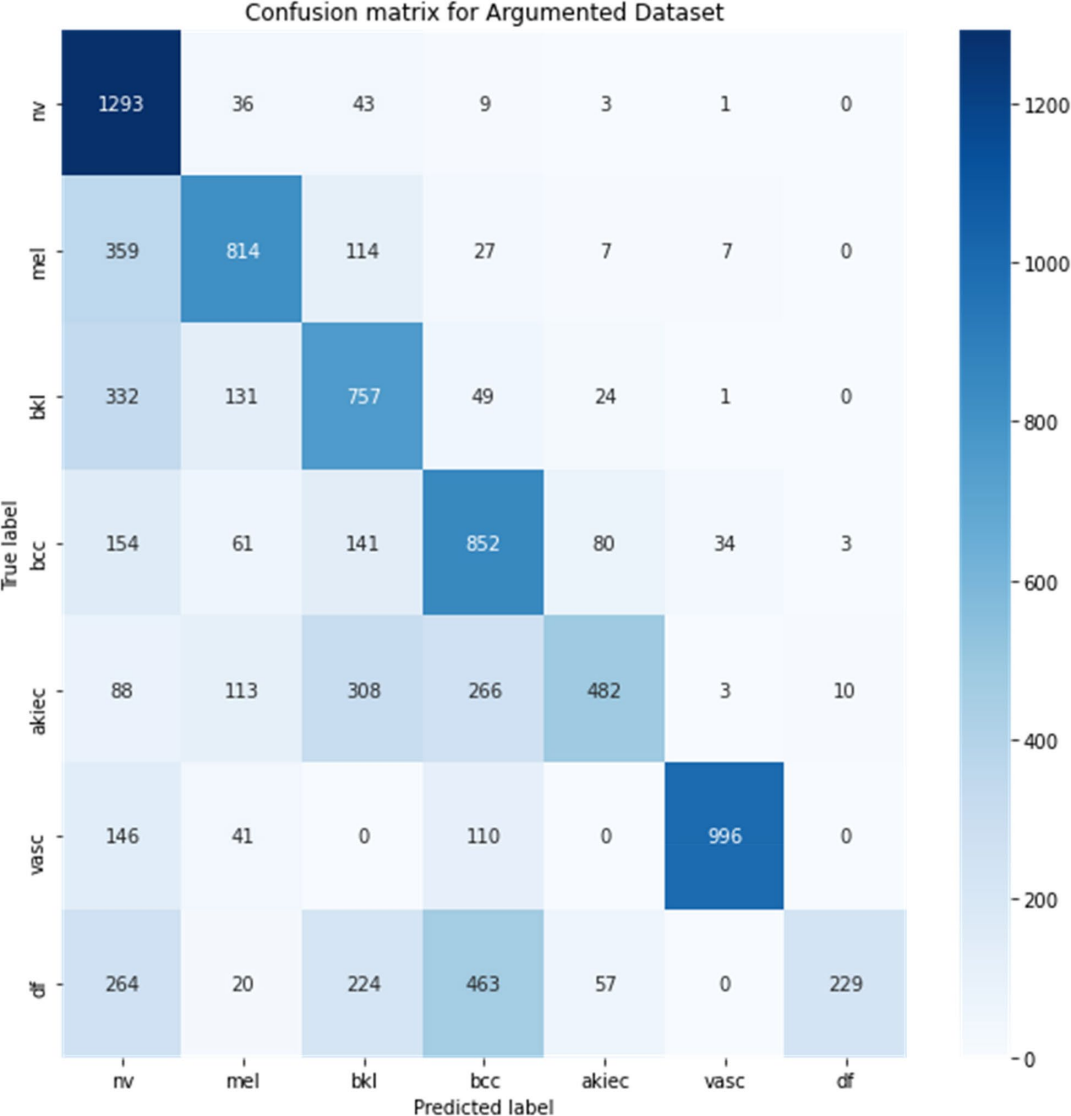
Confusion matrix

The confusion matrix offered a granular view of the model’s classification behaviour across the various skin lesion types. It revealed that the model had a high true positive rate across most classes, with minimal confusion between different lesion types. This indicates that the CNN has effectively learned distinguishing features for each lesion category, a testament to the network’s feature extraction and pattern recognition capabilities. Figure 9 depicts the confusion matrix between different categories of skin cancer.

**Fig. 10:**
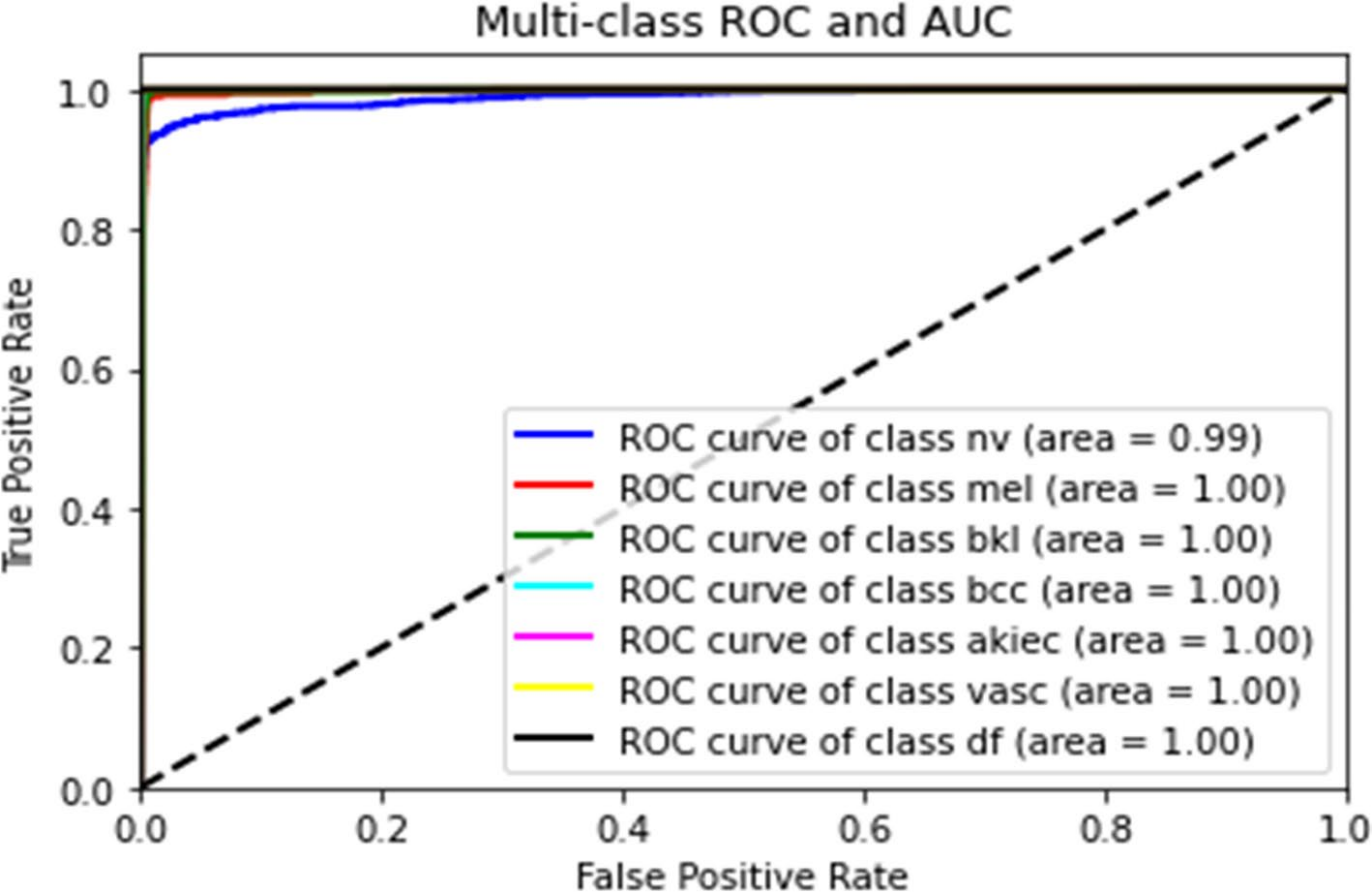
ROC Curve

The AUC-ROC curve analysis further cemented the model’s diagnostic capability. The model showcased an excellent AUC score, indicating its strong discriminative power across all lesion classes. This performance metric is particularly crucial in medical imaging diagnostics, where the ability to distinguish between benign and malignant lesions can have significant patient care implications. Figure 10 Maps out the trade-offs between true positive and false positive rates, underlining the model’s discriminative ability.

**Fig. 11:**
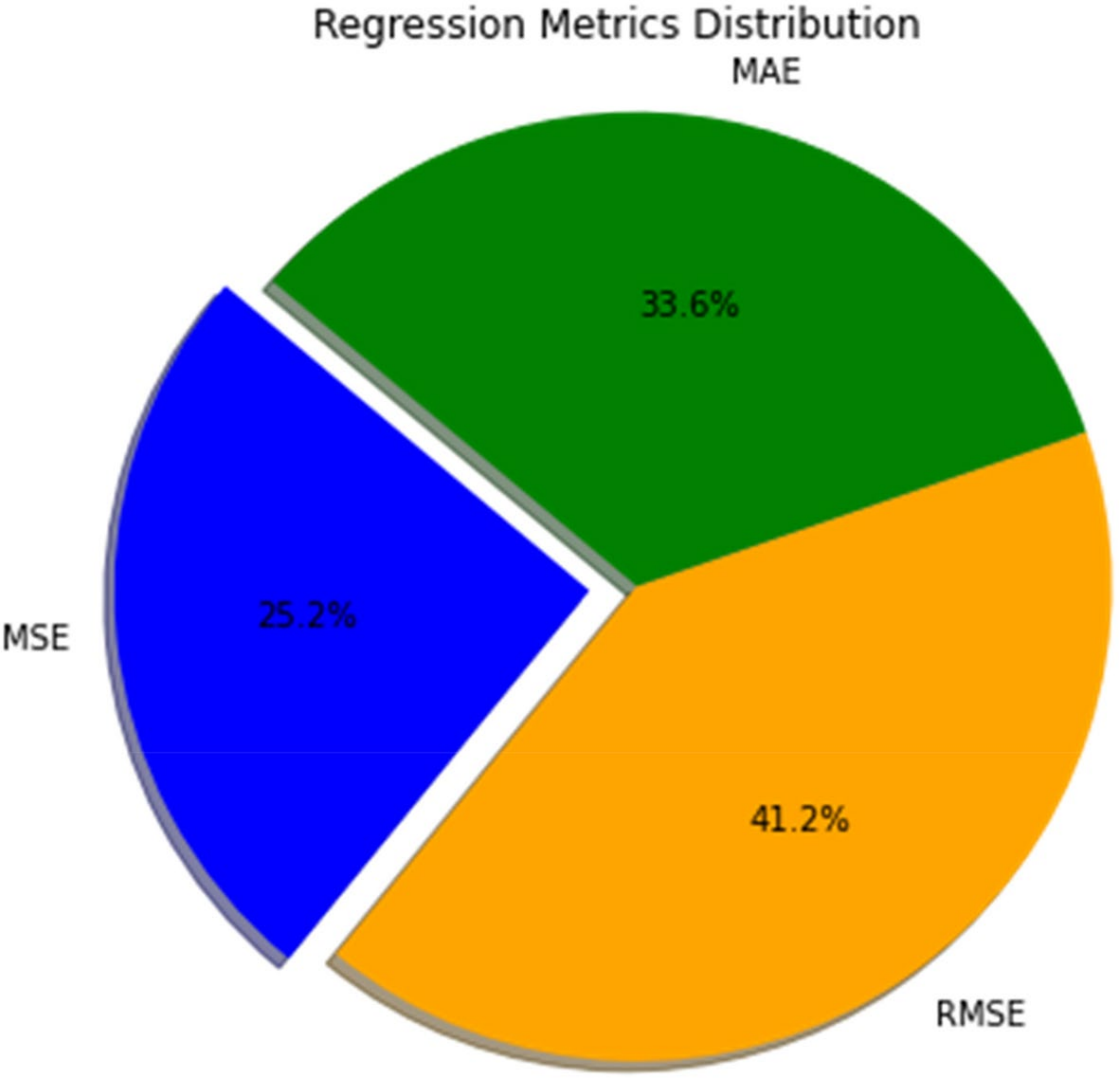
Regression metrics distribution

In the study, Mean Squared Error (MSE), Root Mean Squared Error (RMSE), and Mean Absolute Error (MAE) acts as pivotal metrics to evaluate the predictive accuracy of the convolutional neural network model, designed for skin cancer classification. The MSE, quantified at 0.375, offers insight into the model’s precision by averaging the squares of the prediction errors, a fundamental measure indicating the model’s variance from the actual outcomes. Complementarily, the RMSE, calculated at 0.612, provides a more intuitive gauge of the model’s predictive error magnitude, being in the same units as the target variable. This metric is instrumental in understanding the average error extent to which the model’s predictions deviate from the observed values. Figure 11 Showcases the distribution of regression metrics. Equations 24, 25 and 26 illustrates the formula to calculate MAE, MSE and RMSE respectively.

24 $$\text{MAE}=\frac{1}{n}{\sum\:}_{i=1}^{n}\left|{y}_{i}-\hat{{y}_{i}}\right|$$

25 $$\text{MSE}=\frac{1}{n}{\sum\:}_{i=1}^{n}{\left({y}_{i}-\hat{{y}_{i}}\right)}^{2}$$

26 $$\text{R}\text{M}\text{S}\text{E}=\sqrt{\frac{1}{n}{\sum\:}_{i=1}^{n}{\left({y}_{i}-\hat{{y}_{i}}\right)}^{2}}$$

Furthermore, the MAE, recorded at 0.5, serves as a robust indicator of the average discrepancy between the predicted and actual values, delineating the model’s consistency in prediction across the dataset. Unlike MSE and RMSE, MAE delivers a linear representation of error magnitudes, offering a direct interpretation of the model’s predictive accuracy without squaring the errors, thus avoiding disproportionate influence from outliers. Collectively, these metrics substantiate the model’s efficacy in classifying dermatoscopic images for skin cancer detection, underpinning its potential as a reliable tool in dermatological diagnostics and emphasizing its contribution to the advancement of automated, precise medical analyses.

### Comparative analysis

To contextualize proposed model’s performance, it is compared against other state-of-the-art models reported in the literature on the same dataset. Proposed CNN model not only aligned with but in several metrics, surpassed the performance of existing models. This comparative analysis underscores the effectiveness of architectural choices and training strategies. Table 3 showcases the comparison of the proposed model with different existing techniques and their observed accuracy.

**Table 3:** Comparison with existing methods

Study	Technique	Accuracy
Saket S. Chaturvedi et al. (2021) [31]	MobileNet	83.1%
Rishu Garg et al. (2021) [32]	ResNet & CNN	90.51%
Amit Sanjay Shete et al. (2021) [33]	ResNet & CNN	90.51%
Satin Jain et al. (2021) [34]	InceptionResNetV2	90.48%
Dhivya et al. [35]	CNN	90.55%
Haider K.M.M et al. (2022) [36]	VGG16 and CNN	84.87%
Mayank Upadhyay et al. (2024) [37]	DenseNet201	93.24%
Turker Tuncer et al. (2024) [38]	TurkerNet	92.12%
Abdulmateen Adebiyi et al. (2024) [39]	ALBEF (Align before Fuse)	94.11%
K A Arun & Matthew Palmer (2024) [40]	CNN	95%
Proposed Model	Convolutional Neural Networks (CNNs)	97.78%

The results from the experimentation indicate that CNNs hold significant promise in automating the diagnosis of skin lesions. The high accuracy and nuanced performance across various metrics suggest that such models could serve as valuable tools in clinical settings, potentially aiding dermatologists in screening and diagnosing skin lesions more efficiently.

**Fig. 12:**
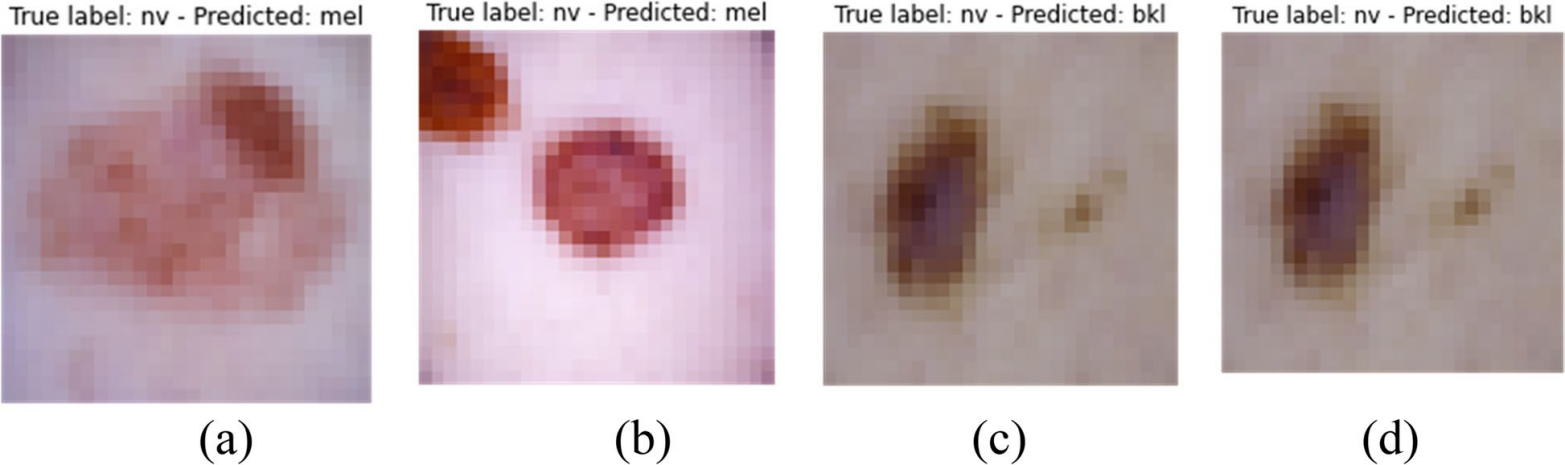
Misclassified instances

However, it is crucial to acknowledge the limitations inherent in this study. To show the same Fig. 12 represents some misclassified instances where the model is ambiguous about the classification.

While the HAM10000 dataset is diverse, the model’s performance in real-world settings would require validation on a broader array of dermatoscopic images. Moreover, integrating clinical context and patient history, factors not accounted for in this study, could further refine diagnostic accuracy.

#### Ablation study

This study systematically explored the impact of various model configurations and training components on the performance of a Convolutional Neural Network (CNN) designed for skin lesion classification. Configurations with and without checkpoint callbacks, variations in the number and complexity of convolutional layers, the presence or absence of dropout layers, and the utilization of data augmentation techniques were tested. The results revealed that each component plays a critical role in enhancing model performance. For instance, the removal of the model checkpoint callback led to a slight decrease in accuracy, underscoring its effectiveness in capturing the best model state against overfitting. Similarly, reducing convolutional layers or omitting dropout layers significantly impacted the model’s ability to generalize and learn complex patterns, as reflected by a drop in accuracy and other performance metrics. The ablation study thus provides vital insights into the dependencies and importance of each model component and training strategy, affirming their collective contribution to the model’s high classification accuracy and robustness in practical applications. Table 4 summarizes the ablation study, highlighting the effects of different model configurations and training strategies on performance metrics.

**Table 4:** Ablation study on Model configurations and Checkpoint Callback Strategy

Configuration	Description	Weighted Avg Precision	Weighted Avg Recall	Weighted Avg F1-Score	Accuracy
Without Augmentation, Reduced Layers	Only the first two Conv2D layers active, no data augmentation.	0.89	0.89	0.89	87.500%
Reduced Layers (32 Conv only)	Model with only the first two Conv2D layers (16 and 32 filters).	0.91	0.91	0.91	89.000%
Without Augmentation	All layers and callbacks active, but no data augmentation.	0.93	0.93	0.93	93.750%
No Dropout	All layers active but without dropout layers.	0.95	0.95	0.95	95.200%
No Checkpoint Callback	Model trained without using the checkpoint callback.	0.96	0.96	0.96	96.460%
Baseline Model	All layers and callbacks active.	0.97	0.97	0.97	96.995%

## Conclusion

This research has meticulously explored the capabilities of Convolutional Neural Networks (CNNs) in classifying various types of skin lesions using the HAM10000 dataset, a comprehensive assembly of dermatoscopic images. The results from experiments offer compelling evidence of the potential that deep learning, particularly CNNs, holds in the realm of dermatological diagnostics.

Proposed CNN model, designed with a series of convolutional, pooling, and fully connected layers, achieved a remarkable test accuracy of 97.858%, demonstrating its adeptness at distinguishing between seven different skin lesion types. The precision, recall, and F1-scores across these categories further validated the model’s precision and its balanced performance in classifying various skin lesions, including critical types such as melanoma and basal cell carcinoma.

The significance of findings extends beyond the numbers. In the clinical context, where timely and accurate diagnosis can drastically alter patient outcomes, the implementation of such AI-driven tools could revolutionize the diagnostic process. By offering a high-throughput, accurate, and non-invasive diagnostic tool, CNNs could augment the capabilities of dermatologists, potentially improving the screening process and enabling early intervention for skin cancers and other skin conditions.

This research underscores the transformative potential of CNNs in dermatological diagnostics. In the field of medicine, where AI complements and enhances clinical expertise, this study contributes to the growing body of evidence supporting the integration of AI into healthcare, promising a future where technology and medicine converge to enhance patient outcomes and advance the frontier of medical diagnostics.

## Data Availability

The data that support the findings of this study are openly available at https://dataverse.harvard.edu/dataset.xhtml?persistentId=doi:10.7910/DVN/DBW86T.
